# Climate-change refugia in the sheltered bays of Palau: analogs of future reefs

**DOI:** 10.1002/ece3.363

**Published:** 2012-08-31

**Authors:** Robert Woesik, Peter Houk, Adelle L Isechal, Jacques W Idechong, Steven Victor, Yimnang Golbuu

**Affiliations:** 1 Department of Biological Sciences, Florida Institute of Technology150 West University Drive, Melbourne, Florida, 32901; 2 Pacific Marine Resources InstituteSaipan, MP, 96950; 3 Palau International Coral Reef Center1 M-Dock Road, P.O. Box 7086, Koror, 96940, Palau; 4 Nature Conservancy, Palau Field OfficeP.O. Box 1738, Koror, 96940, Palau

**Keywords:** Climate change, coral reefs, corals, refugia

## Abstract

Coral bleaching and mortality are predicted to increase as climate change-induced thermal-stress events become more frequent. Although many studies document coral bleaching and mortality patterns, few studies have examined deviations from the expected positive relationships among thermal stress, coral bleaching, and coral mortality. This study examined the response of >30,000 coral colonies at 80 sites in Palau, during a regional thermal-stress event in 2010. We sought to determine the spatial and taxonomic nature of bleaching and examine whether any habitats were comparatively resistant to thermal stress. Bleaching was most severe in the northwestern lagoon, in accordance with satellite-derived maximum temperatures and anomalous temperatures above the long-term averages. *Pocillopora* populations suffered the most extensive bleaching and the highest mortality. However, in the bays where temperatures were higher than elsewhere, bleaching and mortality were low. The coral-community composition, constant exposure to high temperatures, and high vertical attenuation of light caused by naturally high suspended particulate matter buffered the corals in bays from the 2010 regional thermal-stress event. Yet, nearshore reefs are also most vulnerable to land-use change. Therefore, nearshore reefs should be given high conservation status because they provide refugia for coral populations as the oceans continue to warm.

## Introduction

Reef corals are sensitive to increases in water temperature. Corals become pale when temperatures are elevated 1–2°C above average seasonal maximum (Glynn 1991). When high temperatures are sustained for a week or more, the most temperature-sensitive corals such as *Pocillopora, Stylophora*, *Seriatopora*, and *Acropora* will bleach (Hoegh-Guldberg 1999; Edwards et al. 2001; Loya et al. 2001). These bleached corals may subsequently die of starvation because they have lost their symbionts and hence have lost their autotrophic capacity (Glynn 1993; Brown 1997; Fitt et al. 2001). Coral bleaching events are becoming more frequent and are predicted to increase in intensity as the oceans continue to warm (Hoegh-Guldberg 1999; Aronson et al. 2000; Hughes et al. 2003; Hoegh-Guldberg et al. 2007; Baker et al. 2008).

Whether or not coral assemblages will persist through time is, in part, dependent on the frequency and severity of temperature stress. For example, in 1998, reefs in southern Japan that were exposed to temperatures 3°C above the summer average lost nearly 85% of the corals (Loya et al. 2001), and showed major long-term changes in species composition (van Woesik et al. 2011). Whereas, in the same year, nearby reefs exposed to temperatures 1.8°C above the summer average showed only subtle, mortality-induced shifts in size-frequency distributions (Roth et al. 2010). Although the intensity and duration of elevated temperatures are strong predictors of a coral's fate, the extent of bleaching is also dependent on a number of other variables, including: (1) the composition of the coral community (Loya et al. 2001; McClanahan 2004), (2) the seasonal and long-term, “background” temperature (McClanahan and Maina 2003; McClanahan et al. 2007; Thompson and van Woesik 2009), (3) the recent history in exposure to irradiance (Dunne and Brown 2001; Mumby et al. 2001; Brown et al. 2002), (4) the degree of exposure to dissolved inorganic nitrogen concentrations (Wooldridge and Done 2009; Wagner et al. 2010), and (5) water flow rates (Nakamura and van Woesik 2001; van Woesik et al. 2012).

Among the array of bleaching predictors, coral species composition is the strongest (Loya et al. 2001; McClanahan 2004; van Woesik et al. 2011). For example, reefs supporting mainly pocilloporids and acroporids are more likely to bleach than reefs supporting faviids and massive *Porites* (Loya et al. 2001). Given the same species composition, however, the extent of thermal tolerance is also dependent on geographic locality (Thompson and van Woesik 2009). Historical temperature trends show that some reefs, for example, in western Micronesia have previously experienced thermal anomalies at 50-year intervals, whereas the reefs in eastern Micronesia have experienced more frequent thermal anomalies, every 5–6 years (Thompson and van Woesik 2009). It is also becoming clear that localities that have experienced high-return frequencies (5–6 years) over the past several centuries are also experiencing the highest intensities of modern thermal stress (Thompson and van Woesik 2009).

Apart from the spatial heterogeneity of ocean temperatures, local habitat characteristics may also influence the response of corals to thermal stress. For instance, corals in habitats that experience high daily temperature fluctuations are less likely to bleach during regional temperature anomalies than corals in habitats that experience naturally low daily temperature fluctuations (Craig et al. 2001; McClanahan and Maina 2003). Presumably, acclimation to locally dynamic, eurythermal conditions would make corals more tolerant to regional thermal stress.

A more localized parameter that determines the extent to which corals bleach under temperature stress is the intensity of irradiance (Iglesias-Prieto et al. 1992; Brown et al. 2002; Takahashi et al. 2004). Suspended particles in nearshore environments are known to reduce irradiance (Golbuu et al. 2007), which in turn can reduce bleaching susceptibility (Wagner et al. 2010). For example, in Florida, nearshore corals growing in turbid conditions with low irradiance were less prone to bleaching, despite elevated temperatures (Wagner et al. 2010). Notably, however, localities with high nutrient concentrations in the Florida Keys, particularly those with high dissolved inorganic nitrogen concentrations, were more likely to show a bleaching response under anomalously high-temperature events than localities with low-nutrient concentrations (Wagner et al. 2010). A similar response was observed on the Great Barrier Reef, where Wooldridge and Done (2009) suggested that corals in localities with low-water quality, measured as high satellite-derived chlorophyll-a concentrations, were more likely to bleach in 1998 and in 2002 than corals in localities with low chlorophyll-*a* concentrations.

**Figure 1:**
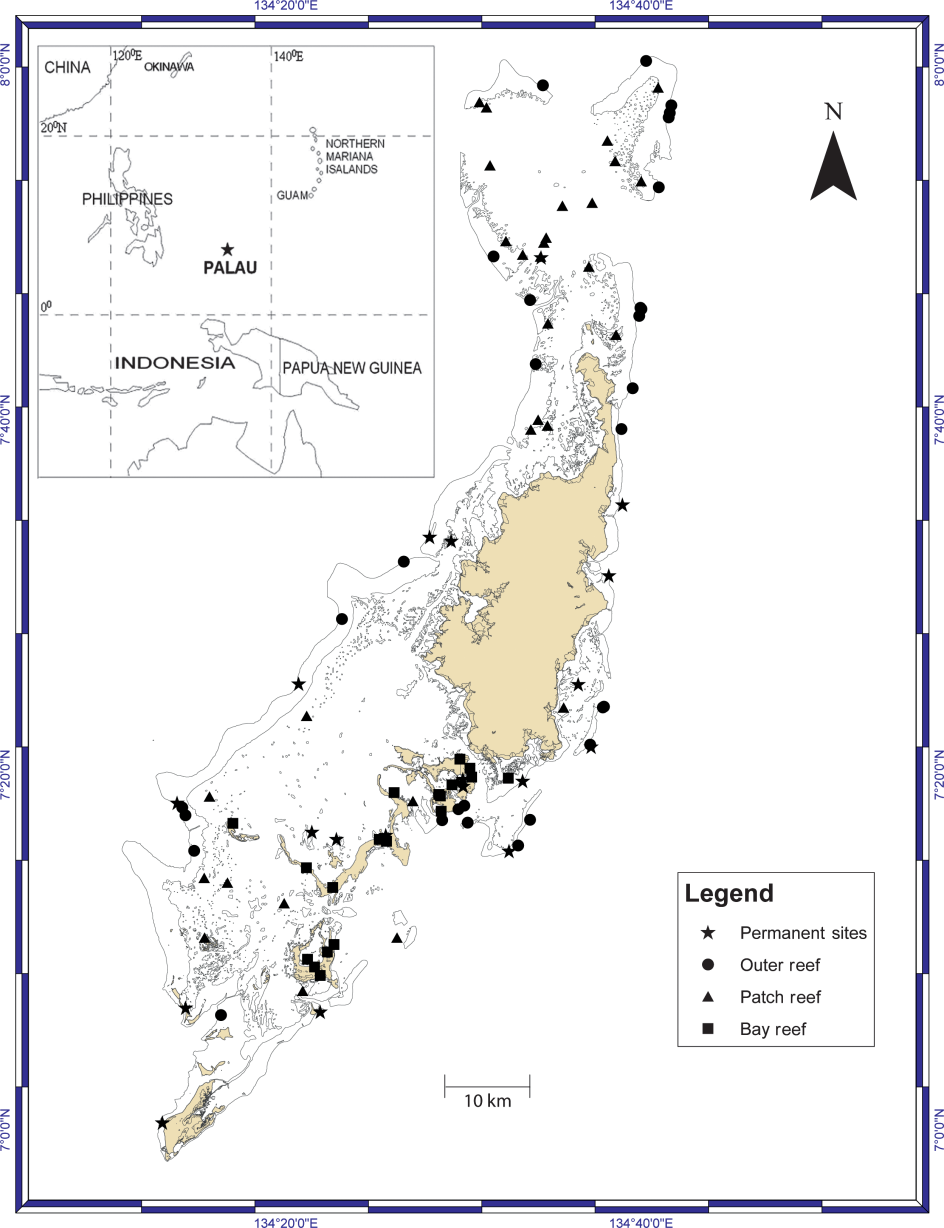
Eighty study sites, stratified by habitat, which included: (1) bays (*n* = 20), (2) patch reefs (*n* = 30), and (3) outer reefs (*n* = 30) that were examined for coral bleaching in July 2010, and the location of the 20 permanent study sites that were analyzed in 2005, 2007, 2009, and 2011 in Palau.

In 1998, the coral populations of Palau, Micronesia suffered a considerable decline during the thermal-stress event that recorded temperatures 1.25°C above the long-term seasonal average (Bruno et al. 2001). However, coral cover recovered to previous levels less than a decade later (Golbuu et al. 2007). In 2010, western Micronesia experienced another thermal-stress event—with satellite-derived sea surface temperatures as high as 1.24°C above average. This study examined the spatial variation and the extent of coral bleaching in Palau during the 2010 thermal-stress event (Fig. 1). The main objectives were to: (1) examine the response of corals to the regional thermal-stress event in different coral-reef habitats, (2) determine the extent to which bleaching patterns were predicted by temperature regimes, and (3) examine which environmental parameters were most likely related to any differential bleaching responses. Our hypothesis is that nearshore reefs in Palau suffer less bleaching during regional thermal-stress events than outer reefs, because they experience higher average temperatures and lower light fields than outer reefs.

## Methods

### Study location and bleaching assessment

This study was undertaken in Palau, western Micronesia (07°30′N, 134°30′E) (Fig. 1). Although the Palau archipelago is about 700 km long, this study focused on the reefs around the main islands (Fig. 1). An assessment of the extent of coral bleaching was undertaken from 28 July 2010 to 12 August 2010. The study employed a stratified-random sampling approach to differentiate the reefs as either: (1) reefs in bays, (2) patch reefs, or (3) outer reefs. Stratification of Palau's reef habitats was based on the 2005, National Oceanic and Atmospheric Administration (NOAA) benthic habitat maps of Palau. The habitat shape files were accessed using Arc 9.3® from which random points were selected, using the Hawth's Analysis Tools for ArcGIS (HawthsTools® 2009), and used as sampling sites. In total, 80 random sites were selected, 30 sites on the outer reefs, 30 sites on the patch reefs, and 20 sites in the bays (Fig. 1). There were no patch reefs surveyed in bays, just fringing reefs. The survey targeted the shallow coral-reef assemblages between 2 and 5 m.

Within each site, 3 × 30-m fiberglass transect tapes were haphazardly placed on the reef within the predetermined depth range (2–5 m below low-water datum). With the camera mounted on a PVC frame, thirty 1-m^2^ contiguous photographs were taken along each transect. The photographs were examined in the laboratory to derive data on bleaching prevalence. Bleaching prevalence was calculated as the percentage of colonies of a particular genus that bleached relative to all the colonies of that genus that were recorded. Each coral colony >1 cm, with its center inside the 1-m^2^ frame, was identified and its longest diameter (assuming an ellipse) measured to the nearest centimeter, and assessed for the percentage of the colony area that was bleached, as either: (1) no bleaching, (2) pale, or (3) bleached. Some coral colonies simultaneously showed all three responses, which were reported as percentages. In total, 34,397 coral colonies were assessed in Palau during the 2010 thermal-stress event.

### Permanent study sites

To determine the extent of coral mortality that was associated with the 2010 thermal-stress event, the pre-existing permanent monitoring sites were re-surveyed for changes in coral cover (Golbuu et al. 2007). Thirteen permanent sites were established in 2001, and an additional seven sites were added by 2005 (Fig. 1). This study used the data from 2005 onward (i.e., 2005, 2007, 2009, and 2011). At each site, five haphazardly placed 50 m × 0.25-m^2^ belt transects were sampled (Golbuu et al. 2007). Although the permanent monitoring sites were depth stratified at 3 and 10 m below low-water datum, this study only examined the 3-m data, which was congruent with the bleaching assessment protocol.

In 2005, 2007, and 2009, an underwater digital video camera (SONY, DCR-PC120, NTSC, with a 0.6× wide lens; Sony Corporation, Tokyo, Japan) was used, in a Sea & Sea VX-PC Underwater Video housing, to record the coral communities. To track changes in coral cover through time, 40 images were systematically extracted from each 50-m video transect, by dividing the recording time of each transect by 40. In 2011, 50 cm by 50 cm still-images were taken every meter along the 50-m transect, using a Sea & Sea model DX-2G camera with Perspex housing (Sea and Sea Products Ltd., Saitama, Japan). A random number generator was used to select 40 of the 50 images that were taken in 2011. For each sampling period, each image was examined for benthic coverage by placing five randomly allocated crosses on a computer screen and then identifying the taxa under each cross to the lowest taxonomic resolution. Two hundred images were analyzed per site, and 4000 images were analyzed per sampling period.

### Environmental data

Ocean sea surface temperatures and chlorophyll-*a* concentrations were collected from the Moderate Resolution Imaging Spectroradiometer (MODIS) satellite sensor maintained by the National Aeronautics and Space Administration. High resolution, processed datasets for the study region are served through the National Oceanic and Atmospheric Administration coast watch program (http://coastwatch.pfeg.noaa.gov/). This study collected MODIS temperature and chlorophyll-*a* datasets using the 0.0125 degree resolution product, or approximately 1.47-km resolution, at 3-day intervals. Data were downloaded as netCDF files and manipulated using Ferret software (http://ferret.wrc.noaa.gov/Ferret/). In order to characterize ocean conditions during the 2010 bleaching event, we constrained our analyses to the summer months (1 May–31 August 2010). We examined: (1) 3-day maximum temperatures, (2) temperature anomalies, calculated by subtracting the mean sea surface temperature (SST) during the study period from the long-term SST mean (2002–2011), (3) the standard deviation of the SST during the study period, (4) chlorophyll-*a* concentration during the study period, and (5) the interaction between mean SST and chlorophyll-*a* concentration.

### Data analysis

To examine differences in coral-community structure among the three habitats, we used a non-metric Multi-Dimensional Scaling (nMDS) analysis, using PRIMER v6.1 (Clarke et al. 2005). The data matrix consisted of mean coral abundance across the three transects at each site. A Bray–Curtis Similarity matrix was created from the square root-transformed data matrix prior to conducting the nMDS analysis. Subsequently, ANOSIM (analysis of similarities) tests were performed to examine if differences in species composition were predictable among habitats (Clarke and Gorley 2001; Clarke and Warwick 2001). Subsequent SIMPER analyses were conducted to examine which coral genus contributed most to the habitat differences.

We used General Linear Models (GLMs) to determine whether there were any differences in bleaching of specific coral taxa among habitats. The prevalence of coral bleaching was examined for violations of normality using normal probability plots, and examined for violations of homogeneity of variances using the Levene's test. To visualize the general bleaching patterns recorded over the sampling space, we spatially interpolated the bleaching-prevalence data for the entire sampling domain using a Kriging function that incorporated Gaussian, best-fit modeling (Wagner et al. 2008).

We also used GLMs to estimate the change in percentage coral cover through time at the 20 permanent monitoring sites. We analyzed the difference in coral cover across sequential samples to overcome potential problems of dependency because of repeated sampling at the same locations. In all instances, the raw data were examined for violations of normality and heterogeneity as described above.

Before conducting regression analyses, we checked for spatial autocorrelation problems, so as not to overestimate the degrees of freedom (Legendre 1993). Spatial autocorrelations were examined using the Moran's I test and treating the latitude and longitude values as if projected on a plane because our study region was geographically constrained (R Core development team 2011; version 2.1). Least-square linear regressions were then conducted to examine whether there were relationships between the prevalence of coral bleaching and the various environmental variables defined above, including the: (1) maximum SST, (2) SST anomalies from the seasonal mean, (3) standard deviation of SST, (4) chlorophyll-*a* concentration, and (5) interaction between SST and chlorophyll-*a* concentration. All analyses were undertaken in R (R Core development team 2011, version 2.1) on a windows platform unless specified.

## Results

Inherent difference in the coral communities existed across the habitats examined. The coral communities in the bays were significantly different from coral communities on the outer reefs (ANOSIM, *R* = 0.513, *p* < 0.01). Bays were dominated by *Porites rus* and *P. cylindrica*, which contributed 41% of the similarity among sites within the bays, whereas outer reefs were defined primarily by encrusting *Montipora*, and branched *Pocillopora* and *Acropora* (which contributed 45% to the similarity). Both bays and outer reefs, however, supported faviids and *Acropora*. The coral communities in bays and patch reefs were significantly (ANOSIM, *R* = 0.0890, *p* < 0.01), but not strikingly (low *R*), different from each other. Similarly, the outer and patch reef communities were significantly different from each other (ANOSIM, *R* = 0.178, *p* < 0.01), although both habitats supported abundant *Montipora* and *Acropora* colonies.

**Figure 2:**
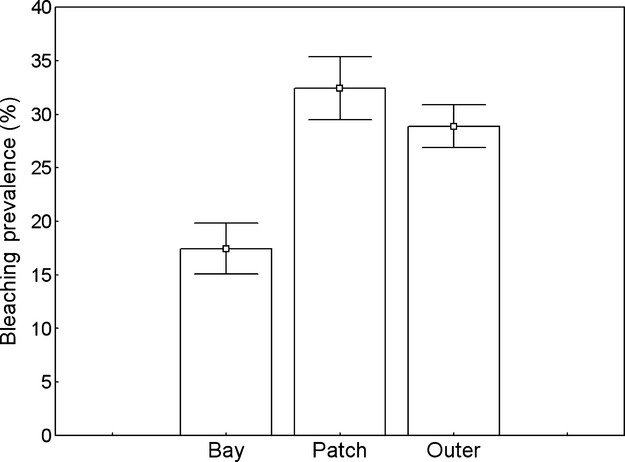
Bleaching prevalence, which is the proportion of colonies that bleached within each population (plus the standard errors), stratified by habitat type, for the thermal-stress event in Palau in 2010.

**Figure 3:**
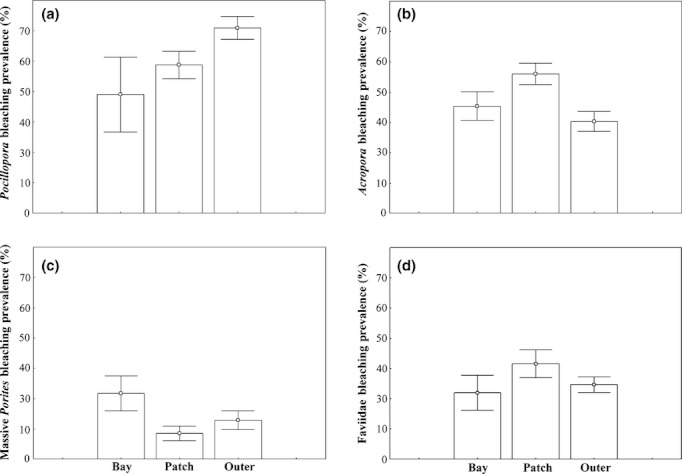
Differences in the prevalence of bleaching (and standard errors) of: (a) *Pocillopora*, (b) *Acropora*, (c) *Porites*, and (d) faviids among habitats.

The earliest sign of coral bleaching in Palau was observed in late June 2010. By mid-July, bleaching was observed in most reefs habitats. Overall, coral bleaching was significantly higher on outer reefs than in bays (*P* < 0.001; Fig. 2, Table 1). *Pocillopora* populations suffered the most extensive bleaching (68%), followed by *Psammocora, Seriatopora* and *Fungia* (Table 1). There were significantly more *Pocillopora* colonies bleached on the outer reefs than in the bays (*P* < 0.001; Fig. 3). *Acropora, Porites*, and faviids showed no significant differences in bleaching among habitats (Fig. 3). *Pocillopora* was the only coral taxa to show a decline between 2009 and 2011, although the decline was not significant. *Acropora* and faviids significantly increased between 2009 and 2011 (*P* < 0.01), and *Porites* showed no significant change through time. There was no relationship between bleaching prevalence and the size of coral colonies for all taxa examined.

**Table 1:** Bleaching susceptibility, defined as prevalence, or percentage of colonies of a particular taxa that bleached, in July–August 2010. Genera with <5 colonies are not shown. The coral genera are displayed from highest to lowest bleaching prevalence on the outer reefs

	Bay reefs		Patch reefs		Outer reefs	
			
Genus	Number of colonies	Bleaching prevalence	Number of colonies	Bleaching prevalence	Number of colonies	Bleaching prevalence
*Pocillopora*	107	14	585	52	1754	68
*Psammocora*	10	30	43	30	32	63
*Seriatopora*	64	25	1196	69	156	62
*Fungia*	363	18	375	41	41	61
*Lobopyllia*	157	34	12	25	7	57
*Platygyra*	47	2	36	53	51	53
*Astreopora*	9	11	161	39	34	47
*Symphyllia*	79	68	11	18	13	46
*Favia*	113	19	138	54	310	45
*Acropora*	544	44	2944	48	5560	37
*Montastrea*	14	7	1	0	9	33
*Favites*	210	17	204	43	528	30
*Galaxea*	7	43	49	14	569	27
*Acanthastrea*	7	14	2	50	16	25
*Stylophora*	1	0	42	57	199	25
*Goniastrea*	357	7	108	41	437	22
*Hydnophora*	5	80	72	57	205	21
*Ctenactis*	58	10	45	20	5	20
*Cyphastrea*	6	0	101	23	127	16
*Leptoria*	5	0	48	15	187	16
*Montipora*	231	13	1074	23	3223	15
*Faviidae*	389	19	1073	17	1245	15
*Echinopora*	49	10	47	36	51	14
*Porites* massive	507	16	578	14	920	11
*Porites* branching	2038	9	1514	18	687	10
*Millepora*	0	0	28	18	103	10
*Merulina*	15	13	13	31	12	8
*Pachyseris*	9	22	21	19	12	8
*Diploastrea*	1	0	2	0	21	5
*Pavona*	200	6	560	8	570	5
*Heliopora*	0	0	17	0	207	2
*Goniopora*	120	6	0	0	15	0
*Heliofungia*	179	0	0	0	0	0
*Pectinia*	14	50	2	50	0	0
*Turbinaria*	1	100	11	9	1	0

**Figure 4:**
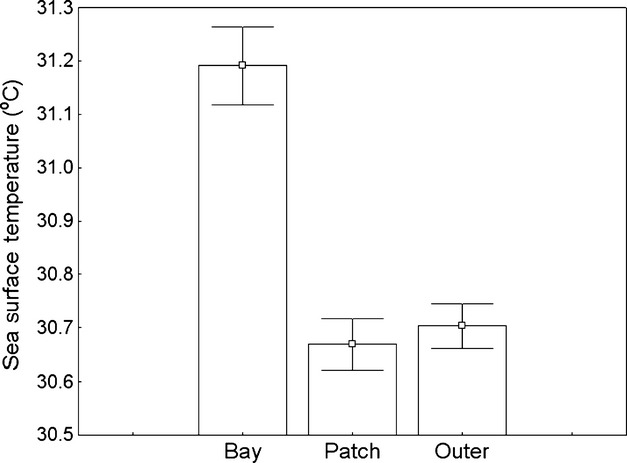
Maximum sea surface temperatures (plus the standard errors) in the three coral-reef habitats in July 2010 (data derived from the MODIS platform that was processed at 3-day intervals and at 0.0125 degree resolution).

**Figure 5:**
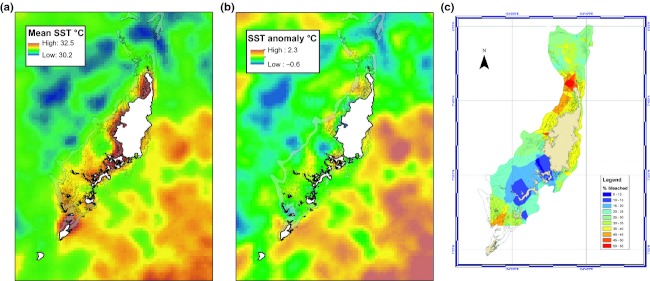
(a) Mean sea surface temperature (SST) for Palau during the 2010 bleaching period: (1 May–31 August 2010) using data derived from the MODIS platform that was processed at 3-day intervals and at 0.0125 degree resolution, (b) SST anomalies for Palau calculated by subtracting the mean SST during the bleaching period from the long-term average (2002–2011), and (c) coral bleaching prevalence in Palau July–August 2010.

The highest temperatures were recorded in the bays (Fig. 4), and the highest temperature anomalies were recorded in the western and northwestern sectors of Palau, and in the bays (Fig. 5). The bays also showed significantly higher variances in temperature than patch and outer reefs between 1 May 2010 and 31 August 2010 (ANOVA, *P* < 0.01). On the outer and patch reefs, coral bleaching was generally coupled with maximum temperature stress and bleaching was particularly severe in the northwestern lagoon of Palau (Fig. 5). The Moran's I was 0.015, suggesting that there was no significant spatial autocorrelation in the bleaching data. The general linear regression models showed that no variable explained more than 10% of the variance in the bleaching-prevalence data, including the satellite-derived data for maximum SSTs, SST anomalies, standard deviation of SST, and chlorophyll-a concentrations. The extent of coral bleaching in 2010 was not related to the size of the coral colonies. The lack of expected relationships was attributed to a decoupling between thermal stress and bleaching prevalence in the nearshore (bays) reefs, where thermal stress was comparatively high, but bleaching and mortality were low (Table 1).

## Discussion

### Thermal stress

In 2010, coral bleaching was particularly severe in the northwestern lagoon, especially on the outer barrier and patch reefs. Bleaching and mortality were, however, low in the bays. *Pocillopora* populations suffered the most extensive bleaching and mortality particularly on the western outer reefs. This is not surprising considering that the genus *Pocillopora* is among the most thermally sensitive corals globally (Glynn 1993; Loya et al. 2001; McClanahan et al. 2004). However, other genera that showed extensive bleaching, including the thermally sensitive *Acropora*, appeared to recover because there was no significant mortality of *Acropora* at the permanent monitoring sites.

Relatively low bleaching and mortality of corals in the bays was likely a consequence of the community composition, and the acclimatization of those communities to constantly high temperatures. The dominant corals in the bays were *Porites rus*, *Porites cylindrica*, and the massive *Porites lobata*, and *Porites australiensis*, which are all known to be among the most resistant corals to thermal stress (Loya et al. 2001; McClanahan 2004). Yet, the branched *Porites cylindrica* in southern Japan was vulnerable to the 1998 thermal-stress event, showing few surviving colonies, whereas bleached *Porites lobata* survived the same event (Loya et al. 2001). The colonies in southern Japan were exposed to an extreme regional thermal-stress event, not dissimilar to the 2010 thermal-stress event in Palau, but the seasonal fluctuations in Japan and Palau are different. The annual SST on reefs in southern Japan ranges from 20°C in winter to 30°C in summer, or approximately 10°C (Bena and van Woesik 2004), whereas the SSTs in the bays of Palau vary by 3.5°C seasonally, from 28 to 31.5°C (Penland et al. 2004). Therefore, we suspect that the constantly high temperatures in the bays of Palau were conducive to thermal acclimatization, whereas the same species in Japan were not acclimatized to high temperatures and suffered extensive mortality during regional thermal stress.

**Figure 6:**
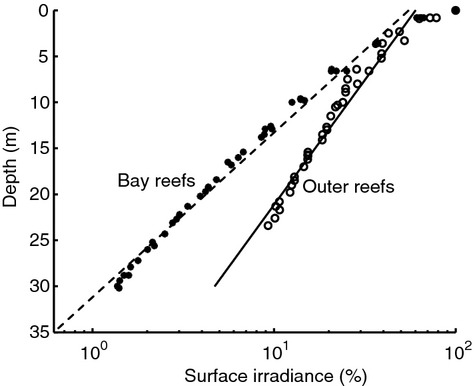
Attenuation of surface irradiance at two habitats, bay reef (Nikko Bay) and outer reef (Short Drop-off) using a Photosynthetically Active Radiation sensor, Biospherical Instrument Inc., Model number, QSP–170. Data were taken on 22 February 2005 during cloud free conditions; the small squares represent field data collected in the bays, and the crosses represent field data collected on the outer reefs. Data were fit to the linear function of the form *E*(*z*) = *E*_o_e^−*kz*^, *E*(*z*) is the irradiance at depth *z*, where *E*_o_ is the irradiance just below the surface, and *k* is the vertical attenuation coefficient that was 0.129 for the bay reef, and 0.085 for the outer reef (modified from Golbuu et al. 2007).

Yet, chronic photoinhibition, or bleaching, is a consequence of the interaction between temperature and irradiance, which both target the reaction centers of the photosynthetic apparatus of symbiotic dinoflagellates (Iglesias-Prieto et al. 1992; Brown et al. 2002; Takahashi et al. 2004). Photoinhibition during bleaching events is caused by excessive irradiance that damages the photosynthetic reaction centers of PSII molecules (Iglesias-Prieto et al. 1992; Takahashi et al. 2004). High-temperature stress simply exacerbates photodamage at those reaction centers. Therefore, low irradiance, caused by naturally high concentrations of suspended particles in Palau's bays, most likely buffered the corals from excessive thermal stress (Iwase et al. 2008; Fig. 6).

Fabricius et al. (2004) argued that high-thermal tolerance of nearshore corals in Palau was a result of the corals supporting thermally tolerant, clade D symbionts. These arguments agree with Baker et al. (2004) who showed more clade D symbionts in corals in the Arabian Gulf where temperature fluctuations were higher than elsewhere in the region. Although it is becoming clear that clade D symbionts are more thermally tolerant than other clades (LaJeunesse et al. 2010), *Porites*, which is the dominant coral on Palau's nearshore reefs, do not harbor clade D symbionts (Fabricius et al. 2004). The high suspended particulate material in the bays may allow corals to derive some metabolic requirements by feeding (Anthony and Fabricius 2000), especially when symbiont-acquired carbon is low while corals are bleached. Yet, Grottoli et al. (2006) showed that the massive coral *Porites lobata*, common in the bays of Palau, do not acquire carbon through heterotrophy. Therefore, we suspect that during regional thermal-stress less bleaching and high survival of corals in bays of Palau must be attributed to parameters other than symbiont types or heterotrophy.

Corals are known to increase their thermal tolerance by either acclimatization of individuals to short-term elevations in temperature (i.e., a phenotypic response), or by adaptation to climate regimes over multiple generations (i.e., differential mortality changes coral genotype frequencies) (Coles and Brown 2003; Portner 2008). Indeed, different biogeographic regions experience temperature “zones” upon which natural selection acts (Craig et al. 2001; Smith-Keune and van Oppen 2006; Thompson and van Woesik 2009; Barshis et al. 2010; van Woesik and Jordan-Garza 2011). For example, the corals in the northern Great Barrier Reef (GBR) experience average temperatures of ∼28.5°C in the warmest months, whereas corals in the southern GBR rarely experience temperatures above 27°C (Smith-Keune and van Oppen 2006; Wooldridge and Done 2009). Therefore, it was no surprise that under a regional temperature stress event in 1998, the northern nearshore reefs of the GBR, which were accustomed to warmer temperatures, showed less bleaching than the southern nearshore reefs of the GBR (Berkelmans and Oliver 1999).

As only alleles experiencing persistent selection pressure may attain high frequency, coral populations in biogeographic regions with high-return frequencies of thermal stress are more likely to undergo rapid directional selection, and adapt to climate change, than coral populations in biogeographic regions with low-return frequencies (Thompson and van Woesik 2009). A recent example from Singapore showed that *Acropora* and *Pocillopora* populations, which are usually highly susceptible to thermal stress, were tolerant to regional thermal stress (Guest et al. 2012). Upon highlighting a mechanism, Guest et al. 2012 posited that the reef corals in Singapore have “adapted and/or acclimatized to thermal stress”. It remains equally unclear whether the coral populations in the bays of Palau have acclimatized to the higher temperatures, or whether they have adapted to the higher temperatures through selection of more thermally tolerant genotypes. The higher light attenuation in the bays than on other reefs complicates the attribution of thermal tolerance to specific parameters. Just as importantly, perhaps, is the concept that acclimatization is an adaptive trait, which is heritable and is therefore open to selective pressure.

### Future ocean climate

Although the maximum temperatures in July 1998 and in July 2010 were similar, we note that the rate of change and the duration of the thermal stress were greater in 1998 than in 2010, and therefore the 2010 event could be described as a moderate thermal stress event for Palau. Climate models from the United Kingdom Hadley Center (HadCM3) predict that by 2040, Micronesia will experience more steady El Niño-like conditions (Donner et al. 2005). By contrast, the Parallel Climate Model (PCM) shows a more uniform warming across the Pacific Ocean (Donner et al. 2005). Both models, however, predict warmer conditions in eastern Micronesia than in western Micronesia. If historical trends persist into the near future, as predicted by both the HadCM3 and PCM models (Donner et al. 2005) and by contemporary trends (Thompson and van Woesik 2009), then the Marshall Islands, Kosrae, and Pohnpei will most likely experience both more intensive and more frequent thermal stresses than localities in western Micronesia, including Palau, Yap, and potentially Chuuk. Nevertheless, it is uncertain to what extent, and for how long, the naturally high temperatures and low irradiance in the bays of Palau will provide refugia for corals under more frequent and severe regional thermal-stress events.

### Conservation

Although the nearshore reefs of Palau may provide a safe haven for some coral species through climate change-induced thermal stress, these nearshore reefs are also more affected by land-use change than the outer reefs (Golbuu et al. 2011). By contrast, outer reefs are more vulnerable to temperature stress events than nearshore reefs, but are also more removed from direct land-use change. Therefore, incorporating reefs from a variety of habitat types into conservation plans, and reducing river runoff to the nearshore reefs becomes even more pertinent in a warming ocean. Conservation plans that optimize strategies against climate-change scenarios should clearly include nearshore reefs. In summary, identifying climate-change refugia is important for at least three reasons: (1) refugia may potentially buffer coral-reef ecosystem against climate change-induced thermal-stress events, (2) the absence of habitat refugia may underestimate migration potential in spatially predictive models (Mosblech et al. 2011), and (3) the absence of refugia may overestimate rates of regional species extinction (Carpenter et al. 2008). In conclusion, this study shows that reefs around bays were more resistant to regional thermal stress than patch and outer reefs. Nearshore reefs in the bays are therefore valuable refuges to buffer coral-reef ecosystems against climate change-induced disturbances.
